# Dravet Syndrome: An Overview

**DOI:** 10.7759/cureus.5006

**Published:** 2019-06-26

**Authors:** Arsalan Anwar, Sidra Saleem, Urvish K Patel, Kogulavadanan Arumaithurai, Preeti Malik

**Affiliations:** 1 Neurology, University Hospitals Cleveland Medical Center, Cleveland, USA; 2 Neurology, University of Toledo, Toledo, USA; 3 Neurology & Public Health, Icahn School of Medicine at Mount Sinai, New York, USA; 4 Neurology, Mayo Clinic Health System, Minnesota, USA; 5 Pediatrics, The Children's Hospital at Montefiore, Bronx, USA

**Keywords:** dravet syndrome, myoclonic epilepsy of infancy, childhood epileptic encephalopathies, anti-epileptic drugs, intractable epilepsy, scn1a gene mutation, sudep

## Abstract

Dravet syndrome (DS), also known as severe myoclonic epilepsy of infancy (SMEI), is one of the rare early childhood intractable epileptic encephalopathies associated with pleomorphic seizure activity, cognitive decline, motor, and behavioral abnormalities. The convulsive seizure is the most common type seen in DS. After the first episode of seizure-like activity, behavioral disorders and cognitive decline are progressive and long-lasting. The most common etiology identified in patients with DS is a de-novo genetic mutation alpha-1 subunit of voltage-gated calcium channel gene (SCN1A). DS is diagnosed clinically and if unclear, genetic testing is recommended. DS treatment options include anti-epileptic drugs and cannabinoids; ketogenic diet therapy and surgical options such as the deep brain and vagal nerve stimulation. Due to drug-refractory epilepsy in DS, many more therapies are being investigated to increase the longevity of patients.

## Introduction and background

Dravet Syndrome (DS) was first described as severe myoclonic epilepsy of infancy (SMEI) by Charlotte Dravet in 1978 and was later renamed Dravet Syndrome in 1989 [1]. It is a rare form of early-onset genetic epilepsy syndrome that manifests as intractable epilepsy and neurodevelopmental delays. DS was included as a part of eight epileptic encephalopathy syndromes, as reported by the International League against Epilepsy (ILAE) task force [2]. The term ‘epileptic encephalopathy’ is a phenomenon described as behavioral deterioration or regression together with cognitive decline due to epileptogenic activity during the brain maturation period [2]. The genetic basis of DS was discovered in 2001 as a mutation in the voltage-gated calcium channel, alpha-1 subunit (SCN1A) gene on chromosome 2q24 [3].

DS evolves with age; after seizure onset during infancy, neurodevelopmental delays progress to severe neurologic disability. During adulthood, it manifests as persistent motor and cognitive dysfunction [4]. Multiple different types of seizures are seen in DS that include convulsive, myoclonic, absence, focal, obtundation status and tonic seizures [5]. Motor system dysfunction manifests as ataxia, tremors, dysarthria, pyramidal, and extrapyramidal signs [6]. Majority of the patients develop cognitive impairment, visual motor integration, visual perception, and executive dysfunctions, along with language impairment [7]. Patients manifest psychiatric disturbances such as aggressiveness, agitation, obsessiveness, preservation, and hoarding behavior [8]. DS patients have an increased mortality rate during infancy but some survive till adulthood. Sudden unexpected death in epilepsy (SUDEP) and status epilepticus are the most common cause of death among DS patients.

## Review

Epidemiology

Three studies reported that the incidence of DS is 1 in 15,000 to 1 in 40,000 [9-11]. They also showed that DS affects male and female in equal proportions [11]. The first seizure episode usually occurs during the first year of life. A study of 471 patients having seizure in the first year of life showed, 3% of them to have DS [12]. Another study showed that 7% of the patients had DS among those with seizure onset during their infancy [13].

Etiology

In 2001, de novo mutation in alpha-1 subunit of voltage-gated calcium channel gene (SCN1A) was identified in seven totally unrelated DS patients [3]. These mutations were located on chromosome 2q24. More than 90% of these mutations were de novo whereas familial mutations such as missense in nature were located only in 5%-10% [3]. There are no identifiable mutations in SCN1A in about 20%-30% of patients with DS. Additional genes that have been identified with DS phenotype patients are PCDH19, GABRA1, STXBP1, CHD2, SCN1B, SCN2A and rarely KCNA2, HCN1, and GABRG2 [14].

The other theory that has been investigated in the pathophysiology of DS is dysfunction of inhibitory neurons. SCN1A gene (Nav1.1) protein product has been the primary voltage-gated sodium channel of these inhibitory neurons. Impairment of Nav 1.1 located in cell bodies and dendrites results in uncontrolled firing from gamma-aminobutyric acid-ergic (GABAergic) neurons [15].

Clinical presentation

Clinical presentation of DS varies with age. Main symptoms are refractory seizures, developmental delay, cognitive impairment and motor dysfunction [16].

Seizures

Presentation of seizures varies with age. Most of the patients are seizure free up to the age of five months and first seizure appears between the age of five to eight months due to any kind of trigger like fever, vaccination, bathing and sometimes in absence of the trigger [17]. First seizure is usually focal or generalized tonic-clonic activity. If performed an electroencephalogram (EEG) during the first seizure activity, it may be either normal for age or may show abnormal theta activity between 4-5hz over the vertex.

After the age of one year, a child can have focal or generalized tonic-clonic epilepsy or absence seizure. It may result in loss of consciousness or altered level of consciousness. The main triggers at this age group are fever, excitement, stress, and lights particularly flash. EEG in half of the patients there is a slowing of EEG activity and in the other half background activity is overtly slow and poorly organized. EEG pattern consisting of generalized spike waves with isolated or brief discharges of fast polyspike waves may be present [18].

Developmental Delay

Developmental delay is noted with the progression of the age. Hypotonia can be detected in the majority of patients around the age of one year. Ataxia is noted when a child starts walking, dysautonomia as variation in sweating or heat and pyramidal signs have a different frequency and variation [19]. Usually, there is no sign of neuro-developmental delay until the start of seizure activity but soon after the first seizure, neuro-developmental delay signs like unsteady gait, language deficit in constructing a sentence, the deficit in fine motor abilities begin and progress shortly thereafter

Behavioral Disturbance

The most common behavioral disturbances are in DS are in the form of autism, attention deficit-hyperactivity disorder (ADHD), aggressiveness, irritability, relational difficulties, and opposition. Motor and cognitive impairment also influence behavioral changes. These features along with cognitive deficit significantly affect social life and adaptive behavior [20].

Diagnosis

DS is clinically diagnosed and, according to the International League Against Epilepsy (ILAE), the following nine clinical characteristics can be determined over time [4,21]:

(1) A family history of epilepsy or febrile seizures

(2) Normal development before seizures onset

(3) Seizure before one year of age

(4) EEG with generalized spike and polyspike waves

(5) Pleomorphic epilepsy (myoclonic, focal, clonic, absence, and generalized seizures)

(6) Focal abnormalities or early photosensitivity

(7) Psychomotor retardation after 24 months

(8) Exacerbation of seizures with increased body temperature

(9) The appearance of subsequent ataxia, pyramidal signs or interictal myoclonus after the beginning of psychomotor slowing.

As per the North American Consensus Panel guideline, genetic testing is recommended in those patients suspected with DS [4,21].

Differential diagnosis

Other infancy/childhood epileptic encephalopathies that must be considered while considering DS are following (Table 1).

**Table 1:** Differential diagnosis considered while considering Dravet syndrome

Syndrome	Age of Onset	Seizure Type	EEG findings
Ohtahara Syndrome [22]	1-3 months	Absence, tonic/clonic, clonic, myoclonic, partial, complex partial seizures	Burst and suppression pattern seen during both wake and sleep state.
West Syndrome (Infantile Spasm) [23]	4-6 months	Epileptic Spasms	Hypsarrhythmia
Doose Syndrome (myoclonic astatic epilepsy) [24]	18-30 months	Focal, myoclonic-atonic and myoclonic seizures	2-3 Hz generalized spike-waves, photo paroxysmal response
Lennox-Gastaut Syndrome [25]	10 day-9 years	Tonic, atypical, myoclonic, focal, generalized tonic-clonic seizure or unilateral clonic seizure	Slow spike-wave complex less than 2.5 Hz frequency and widespread fast paroxysmal activity
Pseudo-Lennox-Gastaut Syndrome (atypical benign partial epilepsy) [26]	18-30 months	Focal, myoclonic-atonic and myoclonic seizures	Sharp multifocal waves, sharp Rolandic waves, epileptic electrical status
Electrical Status Epilepticus during Slow Sleep [27]	2months-12 years	Tonic, atypical, myoclonic, focal, generalized tonic-clonic seizure or unilateral clonic seizure	The normal or slow waves when the patient is awake and continuous spike and wave discharges during deep sleep.
Landau-Kleffner Syndrome [28]	18-months-13 years	Generalized tonic-clonic seizures	Paroxysmal Changes in electroencephalography and little or no development of language
Juvenile Myoclonic Epilepsy [29]	13-19 years	Myoclonic, tonic-clonic and/or absence seizures	The pattern of 3-5 Hz widespread spikes and polyspikes

Management

To treat these patients, various pharmacological and surgical options are now available. Most epileptic encephalopathy patients are resistant to anti-epileptic drugs (AEDs) and most of them have >2 AEDs on board. The first line of drugs and other drugs have been mentioned in Table 2 and Table 3.

**Table 2:** Front line of management a: Agreed upon by moderate consensus; b: Agreed upon by strong consensus; c: Stiripentol not approved for use in all jurisdiction.

Strategy	Options	Comments
First line	Valproic Acid^b^ or Clobazam^b^	If the first choice is not effective, ADD the second line.
Second line	Addition of Stiripentol^bc^ or Topiramate^b^ or Ketogenic Diet^b^	Stiripentol is used in combination with drugs with Valproic acid and Clobazam. The diet for < 2 years is Traditional Ketogenic Diet, 2-12 years is Traditional or Modified Atkins Diet and >12 years is Modified Atkins Diet.
Third line	Clonazepam^b^, Levetiracetam^b^, Zonisamide^b^, Ethosuximide^a^, Phenobarbital^a^ OR Vagus Nerve Stimulation (VNS)^a^	Ethosuximide is for atypical absence seizure. VNS is recommended with evaluation at a Comprehensive Evaluation Center.

**Table 3:** Medications MOA: mechanism of action; GABA: gamma-aminobutyric acid.

Drugs	Starting Dose	Maintenance Dose	Side Effects	Comments
Valproate MOA- Its action is due to blockage of Na+ and Ca++ channels, thus reducing excitability [30]	10 to 15 mg/kg/day in two or three divided doses.	25 to 60 mg/kg/day.	Vomiting, nausea, hair loss, sedation, weight gain, pancreatitis, and blood dyscrasias and hyperammonemia.	Routine monitoring (at baseline and every 6 months) of serum lipase, liver functions, and serum drug level Contraindicated: In pregnancy due to its teratogenic effect.
Topiramate MOA- Its main mechanism of action is due to interaction with the GABA receptor [30]	0.5 to 2 mg/kg/day.	8 to 12 mg/kg/day.	Weight loss, anorexia, renal stones, and behavioral changes.	It has a good safety profile and less interaction with other drugs. A good option when the patient is on several medications.
Stiripentol MOA- It is an allosteric modulator of GABA-A receptor [30]	50 mg/kg per day.	75-100 mg/kg per day.	Decreases appetite and sedation.	It can interact with cytochrome P450 and increase the concentration of other anti-seizure medication.
Levetiracetam MOA- It binds with synaptic vesicle protein 2A and increases GABA [31]	10 mg/kg in two divided doses.	25 mg/kg/dose twice daily	Irritability, depression, and aggression.	It has a minimal drug interaction potential and is generally well tolerated
Cannabidiol MOA- It binds with CB1 and CB2 receptors and counteracts reactive oxygen species [32]	2.5 mg/kg twice daily by mouth.	20 mg/kg per day.	Decreased appetite, diarrhea, somnolence, malaise, and increased transaminase level.	Liver functions must be checked before the start of treatment and after every 3 months.

Ketogenic Diet

The ketogenic diet (KD) has been studied to treat refractory epilepsy since the 1900s [33]. Classic KD consists of long-chain triglycerides, applied in KD ratio of either 4:1 or 3:1 for fat: nonfat (carbohydrates and proteins). The main concept of KD is a high fat, low carbohydrate, and adequate protein diet [33]. The question, as to how KD helps in reducing seizure, is answered on a hypothesis that increases the metabolism of fats leads to increase acetoacetate, which is the main molecule to reduce seizures in mouse models [6]. Studies have shown that KD can reduce up to 50% of seizures in 40% of cases within the first three months [34]. Short-term side effects of KD are intestinal and stomach upset, and mood swings while switching metabolism from carbohydrate to ketone bodies. KD is contraindicated in patients with pancreatitis, hepatic failure, fat metabolism disorders, primary carnitine deficiency, carnitine palmitoyltransferase deficiency, carnitine translocase deficiency, porphyria’s, and pyruvate kinase deficiency [35].

Surgical Therapies

Surgical options for DS are limited. However, deep brain stimulation and vagal nerve stimulators are shown to be effective in decreasing the incidence of breakthrough seizures.

In DBS, patients’ subthalamic or anterior thalamic nucleus is stimulated to decrease the response and seizure frequency but studies on its benefits are limited. In 2018, the U.S. Food and Drug Administration approved DBS as adjunctive therapy for patients with partial seizures that are refractory to three or more AEDs [36].

In vagus nerve stimulation (VNS), the stimulator is placed in the neck and whenever an excess electrical activity is suspected, it sends signals to the vagus nerve to inhibit that activity. It is advised in patients with refractory epilepsy not suitable for surgery, as opposed to or who failed to respond to surgical treatment prior [37].

Utilization of the traditional surgical options like temporal lobectomy and corpus callosotomy can be done after the failure of other treatments options, with considering the potential risk/benefit ratio, disclosed to and discussed with the family before surgery [4].

Support Groups

Multiple support groups exist worldwide to support patients suffering from DS or another resistant epileptic encephalopathy. In the USA, Dravet Syndrome Foundation works with the aim to increase awareness, raise funds and support patients and families [38]. Similarly, in the United Kingdom, Dravet Syndrome UK works with an aim to provide guidance and support patients [39].

Prognosis

Many studies have been conducted to assess the prognosis in DS [40-43]. Two studies have reported that the two most common reason for premature mortality in DS patients is sudden unexpected death in epilepsy (SUDEP) and status epilepticus. It is estimated that 10%-20% of individuals with DS die within 10 years of age [41]. One study by the International Dravet Syndrome Epilepsy Action League (IDEA League) showed that 31/833 DS patients died within 10 years. The mean age of death was 4.6 years, 19/31 of the 31 died due to SUDEP, 10 due to status epilepticus, one due to ketoacidosis and one due to an accident [42]. The average life expectancy of these patients is not well defined. A prospective study of 37 patients showed that both seizure and mental prognosis can be improved in DS patients by preventing status epilepticus occurrence at a young age [43]. Take home message is mentioned in Table 4.

**Table 4:** Take home message EEG: electroencephalogram; SUDEP: sudden unexpected death in epilepsy.

Take Home Message
Dravet syndrome is a distinctive and early-life epilepsy
Diagnosis involves the clinical presentation, imaging, EEG, and genetic testing (Unnecessary testing should be avoided)
Priority must be seizure control including management of status epilepticus, strategies to reduce seizure triggers and prevent SUDEP.
Management consists of antiepileptic medications, ketogenic diet, surgical therapies including vagus nerve stimulation, and cannabidiol/marijuana under strict observation and guidance of the physician
Health care reimbursement, family education, and support groups play a crucial role
Address the comorbidities like gait issues, sleep, gastrointestinal and endocrine issues, dysautonomia and SUDEP.
SUDEP risk reduction can be achieved by seizure detection devices and baby monitoring devices.

Recommendations of the North American consensus panel

Multiple recommendations have been given by the North American consensus panel [4] regarding the diagnosis and management of DS. The panel included 14 expert epileptologists and five parents of children with DS. The recommendations covered all the aspects of the disease, investigation, management, and prognosis. The panel showed a strong consensus on multiple aspects of clinical presentation, type of seizure, seizure triggers, misdiagnosis, neurodevelopment, neurological examination, and family history. Similarly, a strong consensus was also shown regarding multiple aspects of investigations such as MRI (typically normal in patients), EEG findings and genetic testing. In regards to AEDs, clobazam and valproic acid had a strong consensus on being the first line therapy followed by stiripentol and topiramate as second line and levetiracetam as the third option. With strong consensus, the ketogenic diet has been considered as second-line therapy in patients with suboptimal response to clobazam and valproic acid. Surgical therapies such as VNS had a moderate consensus and to be considered after failure of first and second line AEDs. It was recommended not to perform temporal lobectomy in DS patients. Corpus callosotomy can be considered in DS patients with intractable seizure who are not responding to drugs as well as the ketogenic diet.

Regarding genetic counseling strong consensus was shown on educating the family within two to four weeks of the diagnosis by any provider with adequate knowledge about DS. The family must be educated at first visit about risk and management of prolonged seizure, rescue medications, and emergency treatment plan. Risk of death and developmental outcomes should be discussed within four weeks of diagnosis.

Advances and future research

Diagnostic techniques have evolved rapidly from narrow-scale tools (fluorescence in situ and single-gene sequence) to multigene panels, clinical exome sequencing, clinical genome sequencing, and chromosomal microarrays to test infants and children with epilepsy. Similarly, epigenetic biomarkers are playing an important role to improve precision medicine in both genetic and non-genetic pediatric epilepsies. One of the epigenetic factors such as DNA methylation can enhance prognostic progression in DS as they control disease expression and functioning. But due to the limitation on healthcare cost coverage, utilization of diagnostic techniques can be done only if necessary. 

Cannabidiol [44]

A new drug Epidiolex (cannabidiol) for the treatment of refractory epilepsy for patients with DS and Lennox-Gastaut syndrome was approved by the FDA in June 2018. The exact mechanism is only partially understood. It has agonistic activity at cannabinoid receptor 1 (CBD1) and cannabinoid receptor 2 (CBD2) receptors showing anticonvulsant effects. Multiple clinical trials have shown cannabidiol efficacy in patients with DS and it has created a new hope in the DS community to better control seizure frequency and help them to manage their condition effectively.

## Conclusions

DS, an epileptic encephalopathy, is clinically characterized by pleomorphic seizures starting as early as infancy, and exhibit neurodevelopmental delay, cognitive and motor impairment. Like other epileptiform encephalopathies, DS patients are also multi-drug resistant. The first line of the drug is valproate, topiramate, and stiripentol. Other efficacious therapies are cannabinoids and a ketogenic diet. In patient refractory to AEDs and KD, surgical therapies of deep brain stimulation or VNS have been used. Overall, DS has a poor prognosis and need multidisciplinary healthcare management.

## References

[1] Dravet C (2011). The core Dravet syndrome phenotype. Epilepsia.

[2] Khan S, Al Baradie R (2012). Epileptic encephalopathies: an overview. Epilepsy Res Treat.

[3] Claes L, Del-Favero J, Ceulemans B, Lagae L, Van Broeckhoven C, De Jonghe P (2001). De novo mutations in the sodium-channel gene SCN1A cause severe myoclonic epilepsy of infancy. Am J Hum Genet.

[4] Wirrell EC, Laux L, Donner E (2017). Optimizing the diagnosis and management of Dravet syndrome: recommendations from a North American consensus panel. Pediatr Neurol.

[5] Dravet C, Guerrini R (2011). Topics in Epilepsy: Dravet Syndrome. https://www.jle.com/en/ouvrages/e-docs/dravet_syndrome_289598/ouvrage.phtml.

[6] Jansen FE, Sadleir LG, Harkin LA (2006). Severe myoclonic epilepsy of infancy (Dravet syndrome): recognition and diagnosis in adults. Neurology.

[7] Chieffo D, Battaglia D, Lettori D (2011). Neuropsychological development in children with Dravet syndrome. Epilepsy Res.

[8] Martin P, Rautenstraubeta B, Abicht A, Fahrbach J, Koster S (2010). Severe myoclonic epilepsy in infancy - adult phenotype with bradykinesia, hypomimia, and perseverative behavior: report of five cases. Mol Syndromol.

[9] Wu YW, Sullivan J, McDaniel SS, Meisler MH, Walsh EM, Li SX, Kuzniewicz MW (2015). Incidence of Dravet syndrome in a US population. Pediatrics.

[10] Rosander C, Hallbook T (2015). Dravet syndrome in Sweden: a population-based study. Dev Med Child Neurol.

[11] Hurst DL (1990). Epidemiology of severe myoclonic epilepsy of infancy. Epilepsia.

[12] Caraballo R, Cersosimo R, Galicchio S, Fejerman N (1997). Epilepsies during the first year of life [Article in English, Spanish]. Rev Neurol.

[13] Dalla Bernardina B, Colamaria V, Capovilla G, Bondavalli S (1983). Nosological classification of epilepsies in the first three years of life. Prog Clin Biol Res.

[14] Carvill GL, Weckhuysen S, McMahon JM (2014). GABRA1 and STXBP1: novel genetic causes of Dravet syndrome. Neurology.

[15] Chopra R, Isom LL (2014). Untangling the Dravet syndrome seizure network: the changing face of a rare genetic epilepsy. Epilepsy Curr.

[16] Ziobro J, Eschbach K, Sullivan JE, Knupp KG (2018). Current treatment strategies and future treatment options for dravet syndrome. Curr Treat Options Neurol.

[17] Esterhuizen AI, Mefford HC, Ramesar RS, Wang S, Carvill GL, Wilmshurst JM (2018). Dravet syndrome in South African infants: tools for an early diagnosis. Seizure.

[18] Brunklaus A, Ellis R, Reavey E, Forbes GH, Zuberi SM (2012). Prognostic, clinical and demographic features in SCN1A mutation-positive Dravet syndrome. Brain.

[19] Wical B, Leighty D, Tervo M (2009). Signs of dysautonomia in children with Dravet syndrome. Epilepsia.

[20] Ragona F, Brazzo D, De Giorgi I (2010). Dravet syndrome: early clinical manifestations and cognitive outcome in 37 Italian patients. Brain Dev.

[21] Kassaï B, Chiron C, Augier S (2008). Severe myoclonic epilepsy in infancy: a systematic review and a meta-analysis of individual patient data. Epilepsia.

[22] Beal JC, Cherian K, Moshe SL (2012). Early-onset epileptic encephalopathies: Ohtahara syndrome and early myoclonic encephalopathy. Pediatric neurology.

[23] D'Alonzo R, Rigante D, Mencaroni E, Esposito S (2018). West syndrome: a review and guide for paediatricians. Clin Drug Investig.

[24] Stephani U (2006). The natural history of myoclonic astatic epilepsy (Doose syndrome) and Lennox-Gastaut syndrome. Epilepsia.

[25] Filippini M, Boni A, Dazzani G, Guerra A, Gobbi G (2006). Neuropsychological findings: myoclonic astatic epilepsy (MAE) and Lennox-Gastaut syndrome (LGS). Epilepsia.

[26] Hahn A, Pistohl J, Neubauer BA, Stephani U (2001). Atypical "benign" partial epilepsy or pseudo-Lennox syndrome. Part I: symptomatology and long-term prognosis. Neuropediatrics.

[27] Gencpinar P, Dundar NO, Tekgul H (2016). Electrical status epilepticus in sleep (ESES)/continuous spikes and waves during slow sleep (CSWS) syndrome in children: An electroclinical evaluation according to the EEG patterns. Epilepsy Behav.

[28] Deonna TW (1991). Acquired epileptiform aphasia in children (Landau-Kleffner syndrome). J Clin Neurophysiol.

[29] Mahesar S, Akbar HF, Abid H, Sana R (2018). Juvenile myoclonic epilepsy presenting with neurocognitive impairment: a case report. Cureus.

[30] Inoue Y, Ohtsuka Y, Oguni H (2009). Stiripentol open study in Japanese patients with Dravet syndrome. Epilepsia.

[31] Muramatsu K, Sawaura N, Ogata T (2017). Efficacy and tolerability of levetiracetam for pediatric refractory epilepsy. Brain Dev.

[32] Borgelt LM, Franson KL, Nussbaum AM, Wang GS (2013). The pharmacologic and clinical effects of medical cannabis. Pharmacotherapy.

[33] Zhang Y, Xu J, Zhang K, Yang W, Li B (2018). The anticonvulsant effects of ketogenic diet on epileptic seizures and potential mechanisms. Curr Neuropharmacol.

[34] Neal EG, Chaffe H, Schwartz RH (2008). The ketogenic diet for the treatment of childhood epilepsy: a randomised controlled trial. The. Lancet Neurol.

[35] Masood W, Uppaluri KR (2019). Ketogenic Diet. https://www.ncbi.nlm.nih.gov/books/NBK499830/.

[36] Loddenkemper T, Pan A, Neme S (2001). Deep brain stimulation in epilepsy. J Clin Neurophysiol.

[37] Ali R, Elsayed M, Kaur M, Air E, Mahmood N, Constantinou J, Schwalb J (2017). Use of social media to assess the effectiveness of vagal nerve stimulation in Dravet syndrome: a caregiver's perspective. J Neurol Sci.

[38] (2009). Dravet Syndrome Foundation. https://www.dravetfoundation.org/.

[39] (2010). Dravet Syndrome UK. https://www.dravet.org.uk/.

[40] Sakauchi M, Oguni H, Kato I (2011). Mortality in Dravet syndrome: search for risk factors in Japanese patients. Epilepsia.

[41] Shmuely S, Sisodiya SM, Gunning WB, Sander JW, Thijs RD (2016). Mortality in Dravet syndrome: a review. Epilepsy Behav.

[42] Skluzacek JV, Watts KP, Parsy O, Wical B, Camfield P (2011). Dravet syndrome and parent associations: the IDEA League experience with comorbid conditions, mortality, management, adaptation, and grief. Epilepsia.

[43] Akiyama M, Kobayashi K, Yoshinaga H, Ohtsuka Y (2010). A long‐term follow‐up study of Dravet syndrome up to adulthood. Epilepsia.

[44] McCoy B, Wang L, Zak M (2018). A prospective open‐label trial of a CBD/THC cannabis oil in dravet syndrome. Ann Clin Transl Neurol.

